# The reclassification of neurodevelopmental disorders in ICD-11

**DOI:** 10.1007/s00115-025-01876-w

**Published:** 2025-10-01

**Authors:** L. Tebartz van Elst, Andreas Riedel, Monica Biscaldi-Schäfer

**Affiliations:** 1 Department of Psychiatry and Psychotherapy, Medical Center—University of Freiburg, Faculty of Medicine, University of Freiburg, Hauptstraße 5, 79104 Freiburg, Switzerland; 2 Outpatient Services, Lucerne Psychiatric Clinics, Lucerne, Switzerland; 3 Department of Child and Adolescent Psychiatry, Psychotherapy, and Psychosomatics, Medical Center—University of Freiburg, Faculty of Medicine, University of Freiburg, Freiburg, Germany

**Keywords:** Developmental disorders, Autism spectrum disorder, Attention deficit hyperactivity disorder, Tic disorders, Disorders of intellectual development, Entwicklungsstörungen, Autismusspektrumstörung, Aufmerksamkeitsdefizit‑/Hyperaktivitätsstörung, Tic-Störungen, Störungen der Intelligenzentwicklung

## Abstract

In the ICD-10 the developmental disorders are categorized under three different chapters: F7 for mental retardation, F8 for developmental disorders and F9 for disorders with onset in childhood and adolescence. In ICD-11 neurodevelopmental disorders represent the first new main classification group. The disorders grouped in these categories are all characterized by essentially genetically related atypical patterns of perception, emotional processing, cognition (general and social), language and motor skills. These patterns of mental functioning usually manifest in the first decade of development. They represent persisting characteristics of mental functioning, which can be understood as structural diagnoses. As such, they do not always have pathological significance but must be understood as variants of the norm, at least in less severe cases. In such constellations, they often form the psychodynamic basis for characteristic patterns of interpersonal relationship and communication problems in a subsyndromic expression and hinder the development of valid and constructive identities. These psychodynamics are often associated with interpersonal problems and conflicts as well as classical psychiatric comorbidities, such as stress reactions, adjustment disorders, anxiety disorders, obsessive-compulsive disorders, depression, personality disorders or impulsive and psychotic states. Developmental disorders have a high degree of overlap and comorbidity. This article summarizes the conceptual changes in ICD-11 compared to ICD-10, particularly with respect to the four main subgroups: intellectual disability, autism spectrum disorders, ADHD and tic disorders.

## Abstract (de)

Die Entwicklungsstörungen sind bislang in der International Statistical Classification of Diseases and Related Health Problems 10 (ICD-10) in drei Kapiteln konzeptionalisiert (F7, F8, F9). In der ICD-11 stellen sie die erste neue klassifikatorische Hauptgruppe dar. Die in dieser Kategorie zusammengefassten Störungsbilder sind alle durch wesentlich genetisch bedingte musterhafte Besonderheiten der Wahrnehmung, Emotionsverarbeitung, allgemeinen und sozialen Kognition, Sprache und Motorik gekennzeichnet, die sich im Regelfall bereits in der ersten Lebensdekade zeigen. Es handelt sich dabei um anhaltende Besonderheiten des mentalen Funktionierens, die auch als Strukturdiagnosen verstanden werden können, aber nicht immer zwingend Krankheitswert besitzen. Sie bilden auch in einer subsyndromalen Ausprägung häufig die psychodynamische Grundlage für sich darauf aufbauende Probleme im zwischenmenschlichen Miteinander, der Kommunikation, der Entwicklung eines stabilen Selbst sowie einer validen und integrierten Identität. Dies ist häufig mit einer hohen Rate an interpersonellen Problemen und Konflikten sowie mit klassisch psychiatrischen Komorbiditäten wie Belastungsreaktionen, Anpassungsstörungen, Angst- und Zwangsstörungen, Depressionen, Persönlichkeitsstörungen oder impulsiven und psychotischen Zustandsbildern vergesellschaftet. Entwicklungsstörungen zeigen untereinander eine hohe Überlappung und Komorbidität. In diesem Beitrag werden die Änderungen vergleichend zur ICD-10 (vertieft für die 4 großen Untergruppen: Störung der Intelligenzentwicklung, Autismusspektrumstörung, Aufmerksamkeitsdefizit‑/Hyperaktivitätsstörung und Tic-Störungen) zusammengefasst.

## Introduction

Developmental disorders (DDs) represent the first main nosological category in the 11th revision of the International Statistical Classification of Diseases and Related Health Problems (ICD-11), preceding all other psychiatric–psychosomatic disorder entities [2]. By contrast in ICD-10 they were grouped in three different chapters: F7 on mental retardation; F8 on disorders of psychological development; and F9 on behavioral and emotional disorders with onset usually occurring in childhood and adolescence (hyperkinetic disorders and/or attention deficit hyperactivity disorder [ADHD] and tic disorders; [3]).

This reorganization is convincing not only because these entities are often associated with one another, but also because they all represent typical patterns of perceptual and emotional information processing, general and social cognition, language, and motor skills, which, upon closer inspection, already manifest themselves in the first decade of life. They are structural constants throughout life, which is why the concept of a “structural diagnosis” has been proposed [4–6].

Even in milder forms (broader autism/ADHD/tic phenotype), the particularities of personality functioning manifest early in life and are experienced as “being different.” However, the exact nature of this difference is often misunderstood by those affected as well as by their relatives and caregivers. This lack of understanding of their own nature and how they differ from their peers often leads to typical patterns of relationship problems, conflict patterns, and problematic behaviors. These then form the psychodynamic background for disturbances in the development of the self (e.g., self-image, self-esteem, and identity development), which can be accompanied by frustration, feelings of lack of acceptance and recognition, and states of tension, anxiety, and depression. Aggressive and psychotic decompensations may also occur. The latter usually take center stage at the time of referral to the psychiatric–psychotherapeutic support system. If the underlying structural diagnoses are not recognized—which is often the case, especially in mild and subsyndromal forms—the psychodynamics and causality of the resulting psychological symptoms cannot be properly understood. Essential elements of symptom dynamics are overlooked in therapy planning. Therefore, it is important to be familiar even with milder forms of ES, although they are not necessarily understood as diseases in themselves (see Table 1).^1^

**Table 1:** Classification of various developmental disorders (DDs) according to ICD-11 in comparison with ICD-10 and DSM‑5 (modified from [1])

DDs according to ICD-11		DDs according to ICD-10		DDs according to DSM‑5
6A00	Disorders of intellectual development	F7	Mental retardation F70–F79	Intellectual disability
6A01	Developmental speech or language disorders	F80, F98.5, F98.6	Specific developmental disorders of speech and language, stuttering (stammering), cluttering	Communication disorders
6A02	Autism spectrum disorder (ASD)	F84	Pervasive developmental disorders	Autism spectrum disorder
6A03	Developmental learning disorder	F81	Specific developmental disorders of scholastic skills	Specific learning disorder
6A04	Developmental motor coordination disorder	F82	Specific developmental disorder of motor function	Motor disorders
6A05	Attention deficit hyperactivity disorder	F90	Hyperkinetic disorders	Attention-deficit/hyperactivity disorder
6A06	Stereotyped movement disorder	F98.4	Stereotyped movement disorders	Stereotypic movement disorder
6E60	Secondary neurodevelopmental syndrome	–	–	–
6A0Y	Other specified neurodevelopmental disorders	F88	Other developmental disorders	Other specified developmental disorders
6A0Z	Neurodevelopmental disorders, unspecified	F89	Unspecified developmental disorders	Unspecified neurodevelopmental disorders
8A	Movement disorders			*Motor disorders*
8A05.0	Primary tics or tic disorders	F95	Tic disorders	Tourette’s disorder Persistent motor or vocal tic disorder

*DSM* Diagnostic and Statistical Manual of Mental Disorders, *ICD* International Statistical Classification of Diseases and Related Health Problems

## 6A00 disorders of intellectual development according to ICD-10 F7

The term “intellectual disability” (ICD-10) is replaced by the term “intellectual development disorder.” As before, a distinction is made between the two core areas of impaired intellectual performance and impaired adaptive skills, with greater emphasis now being placed on the heterogeneity of impairment profiles and the impairment of functions (cf. International Classification of Functioning, Disability, and Health [ICF]; [7]). The classification into mild (~ IQ 55–69), moderate (~ IQ 40–54), and severe (~ IQ < 40) is retained, with emphasis on the fact that objective quantification is hardly possible in the very low IQ range [8]. The “most severe intellectual disability” according to ICD-10 (estimated IQ < 20) is now referred to as profound intellectual disability (for more details, see AWMF S2k guidelines; [7, 8]).

## 6A02 autism spectrum disorder according to ICD-10 F84.-

Autism spectrum disorder (ASD) largely follows the changes already outlined in the fifth edition of the Diagnostic and Statistical Manual of Mental Disorders (DSM‑5; [9, 10]). The ICD-10 autism subcategories (early childhood autism, F84.0; atypical autism, F84.1; and Asperger syndrome, F84.5) have been replaced by the term “autism spectrum disorder.” The core autistic syndrome has been restructured into two main criteria: deficits in social communication and interaction; and restrictive, repetitive patterns of behavior, interests, activities, and patterns of perception. Compared to the ICD-10, greater weight is given to the second main criterion, which is now also supplemented by sensory characteristics as a qualitative feature. The operationalization of syndromal ASD is less strict than in the DSM‑5. No defined number of symptoms is required for the main criteria, and only symptomatic examples are listed. Unlike in the DSM‑5, severity is not operationalized. Specific reference is made to the phenomenon of compensation. This means that symptoms such as lack of eye contact can be concealed so well through targeted practice, especially in cases of high intelligence, that they go unnoticed for a long time. Pragmatic language deficit symptoms are clearly emphasized.

Furthermore, frequent comorbidities within the ES, such as those between ASD and ADHD, are now more clearly recognized. While the ICD-10 still excluded dual diagnoses of ASD and ADHD, they can now be recognized as comorbid conditions.

Gender-related aspects are also emphasized more strongly. For example, women and girls with autism often have better social skills, are less conspicuous, and show less expansive behavior. This means they are often not recognized or diagnosed. This can have very negative consequences [5, 11], a point that is now underscored more strongly. Finally, the distinction between the spectrum and other mental disorders, and the spectrum of normality in particular, is clearer. Subsyndromal variants of ASD, in the sense of structural diagnoses as normal variants (“broader autism phenotype”), have long been recognized in the specialist literature [4]. Despite being less severe, these can be highly significant, particularly in psychotherapy, in helping to understand interpersonal problems and conflicts as well as the resulting adjustment disorders, anxiety disorders, depression, or personality disorders that can develop ([4, 6]; Table 2).

**Table 2:** Classification of autism spectrum disorders (ASDs) in ICD-11, ICD-10, and DSM‑5 (modified from [1])

ASD according to ICD-11		ASD according to ICD-10		ASD according to DSM‑5
6A02	Autism spectrum disorder	F84	Pervasive developmental disorders	Autism spectrum disorder
6A02.0	Autism spectrum disorder without disorder of intellectual development and with mild or no impairment of functional language	F84.0, F84.1, F84.5	Childhood autism, atypical autism, Asperger’s syndrome	*Additional codes for:* severity, accompanying intellectual impairment, accompanying language impairment, connection with a known physical illness, genetic or environmental condition, comorbidities
6A02.1	Autism spectrum disorder with disorder of intellectual development and with mild or no impairment of functional language			
6A02.2	Autism spectrum disorder without disorder of intellectual development and with impaired functional language			
6A02.3	Autism spectrum disorder with disorder of intellectual development and with impaired functional language			
6A02.5	Autism spectrum disorder with disorder of intellectual development and with absence of functional language			
6A02.Y	Other specified autism spectrum disorder	F84.8	Other pervasive developmental disorders	–
6A02.Z	Autism spectrum disorder, unspecified	F84.9	Pervasive developmental disorders, unspecified	–

*DSM* Diagnostic and Statistical Manual of Mental Disorders, *ICD* International Statistical Classification of Diseases and Related Health Problems

## 6A05 attention deficit hyperactivity disorder

As with ASD, the far-reaching changes to the DSM‑5 with regard to attention deficit hyperactivity disorder (ADHD) are essentially being adopted. The ICD-10 terminology of hyperkinetic disorder of social behavior or simple activity and attention disorder is replaced by ADHD. While the ICD-10 classification focused almost exclusively on children and adolescents, the new classification also takes adults into account. Hyperactivity symptoms, which are more prevalent in childhood and adolescence, are now more firmly embedded within a broader range of symptoms. Compared to the DSM‑5, the criteria for diagnosing ADHD are vaguer and less clearly defined, potentially resulting in more diagnoses. While the age criterion was 5 years in the ICD-10, the critical time window has now been raised to 12 years. Furthermore, as with ASD, diagnoses are permitted in which symptoms only become apparent when social demands exceed the compensatory potential of affected individuals. This allows for a broader and later initial diagnosis, which will lead to an increase in prevalence. Overall, the clinical phenotype description in ICD-11 is much more comprehensive and detailed. It also addresses the difficulty of distinguishing ADHD from other mental disorders and from normal variants (“broader ADHD phenotype”), as well as the differences between boys/men and girls/women [4]. In contrast to the DSM‑5, the severity of ADHD is not operationalized in the ICD-11 ([2, 9]; Table 3).

**Table 3:** Classification of attention deficit hyperactivity disorder (ADHD) in ICD-11, ICD-10, and DSM‑5 (modified from [1]).

ADHD according to ICD-11		ADHD according to ICD-10		ADHD according to DSM‑5
Code	Terminology	Code	Terminology	Terminology
6A05	ADHD	F90	Hyperkinetic disorders	ADHD
6A05.0	Attention deficit hyperactivity disorder, predominantly inattentive presentation	F90.0	Disturbance of activity and attention	ADHS, predominantly inattentive presentation
6A05.1	Attention deficit hyperactivity disorder, predominantly hyperactive-impulsive presentation			ADHS, predominantly hyperactive/impulsive presentation
6A05.2	Attention deficit hyperactivity disorder, predominantly combined presentation			ADHS, combined presentation
6A05.Y	Attention deficit hyperactivity disorder, other specified presentation	F90.1, F90.8	Hyperkinetic conduct disorder, other hyperkinetic disorders	Other specified ADHD
6A05.Z	Attention deficit hyperactivity disorder, presentation unspecified	F90.9	Hyperkinetic disorders, unspecified	Unspecified ADHD

*DSM* Diagnostic and Statistical Manual of Mental Disorders, *ICD* International Statistical Classification of Diseases and Related Health Problems

The strong link between ADHD and common problem behaviors such as drug use, addiction, unstable relationships, and accidents [4, 12] is emphasized, as is its high comorbidity with ASD, tic disorders, obsessive-compulsive disorders, bipolar affective disorders, and epilepsy [2].

## 8A05.0 primary tics or tic disorder according to ICD-10: F95

In the case of tic disorders (TD), a fundamentally new classification has been established in the ICD-11: They are now classified as neurological disorders rather than psychiatric disorders [13]. However, they are listed in the DD chapter as a related category under a neurological code (see Table 4). In the DSM‑5, however, TS are classified as a DD.

**Table 4:** Classification of tic disorders in ICD-11, ICD-10, and DSM‑5 (modified from [1])

Tic disorders according to ICD-11		Tic disorders according to ICD-10		Tic disorders according to DSM‑5
8A05.0	Primary tics or tic disorders	F95	Tic disorders	Tic disorders
8A05.00	Tourette syndrome	F95.2	Combined vocal and multiple motor tic disorder (Tourette syndrome)	Tourette disorder Persistent (chronic) motor or vocal tic disorder
8A05.01	Chronic motor tic disorder	F95.1	Chronic motor or vocal tic disorder	Persistent (chronic) motor or vocal tic disorder
8A05.02	Chronic phonic tic disorder			
8A05.03	Transient motor tics	F95.0	Transient tic disorder	Transient tic disorder
8A05.0Y	Other specified primary tics or tic disorders	F95.8	Other tic disorders	Other tic disorders
8A05.0Z	Primary tics or tic disorders, unspecified	F95.9	Tic disorder, unspecified	Tic disorder, unspecified
*8A05.1*	*Secondary tics*			

*DSM* Diagnostic and Statistical Manual of Mental Disorders, *ICD* International Statistical Classification of Diseases and Related Health Problems

Regarding terminology, the term “phonic tics” is now used instead of “vocal tics.” However, there has been no fundamental change in the description of the phenotype. Specific reference is made to the frequent co-occurrence of TD with other DDs, such as ADHD or ASD, as well as with other mental health conditions, including obsessive-compulsive disorders. The distinction between primary TD and secondary TD, where at least probable etiological or pathogenetic factors can be identified, continues to be made [13, 14].

## 6E60 secondary neurodevelopmental syndrome

In the ICD-11 classification system, all DDs are divided into primary idiopathic and secondary variants. The latter are listed in a separate chapter (6E60). In the case of DD in particular, specific genetic syndromes (e.g., 22q11 and fragile X) and acquired conditions (e.g., inflammatory or immunological central nervous system diseases and fetal alcohol syndrome) can be identified as possible, probable, or certain causes of ES. In such cases, they would be classified as secondary disorders according to the ICD-10 category of organic mental disorders (ICD-10 F0). For more information on specific diagnostic and therapeutic approaches, please refer to the comprehensive literature [14].

## Practical conclusion

- Compared to the ICD-10, the ICD-11 introduced fundamental changes and reorganizations to the classification of developmental disorders (DDs).
- In terms of content, these were largely prepared by corresponding reclassifications in the DSM‑5.
- Developmental disorders are described as patterns of perception, intellectual development, social cognition, emotionality, language and motor skills, as well as learning and behavioral organization, which deviate from the average norm. If the presentation is fully syndromic, they are usually diagnosed in the first decade of life.
- They are persistent in nature, although they can often be compensated for so effectively by learning strategies in cases of good intellectual development that they are less noticeable.
- The dimensional conceptualization of the ICD-11 (based on the DSM-5) places greater emphasis on transitional forms of all DD. These are usually less pronounced and are often subsyndromal phenotypes (“broader autism/ADHD/tic phenotype”).
- In these cases, the diagnosis is often not made initially. However, such structural diagnoses, in the sense of nonpathological nosological entities, can still be relevant for the development of interpersonal problems and conflicts and subsequent mental disorders, such as acute stress reactions, adjustment disorders, depressive episodes, obsessive-compulsive disorders, disorders of self and identity development, and personality development disorders, as well as for therapy planning.
- Knowledge of such syndromal and subsyndromal DDs is therefore important not only in child and adolescent psychiatry and psychotherapy, but also in adult psychiatry, psychotherapy, and psychosomatics, because a comprehensive understanding of the genesis and psychodynamics of many psychological maldevelopments, conditions, and chronic conflict situations cannot be achieved without considering these structural particularities of a person.

## Footnotes

1 The content, structure, and text of this technical article are based on passages written by the first author on this topic (Tebartz van Elst et al. [1]). The translation into English was performed by the first author using free web-based translation tools (Google Translate, DeepL).

## Declarations

For this article no studies with human participants or animals were performed by any of the authors. All studies mentioned were in accordance with the ethical standards indicated in each case.

The supplement containing this article is not sponsored by industry.
